# Purinergic Activation of Ca^2**+**^-Permeable TRPV4 Channels Is Essential for Mechano-Sensitivity in the Aldosterone-Sensitive Distal Nephron

**DOI:** 10.1371/journal.pone.0022824

**Published:** 2011-08-05

**Authors:** Mykola Mamenko, Oleg Zaika, Min Jin, Roger G. O'Neil, Oleh Pochynyuk

**Affiliations:** Department of Integrative Biology and Pharmacology, The University of Texas Health Science Center at Houston, Houston, Texas, United States of America; University of Oldenburg, Germany

## Abstract

Mechanical forces are known to induce increases of [Ca^2+^]_i_ in the aldosterone-sensitive distal nephron (ASDN) cells to regulate epithelial transport. At the same time, mechanical stress stimulates ATP release from ASDN cells. In this study, we combined ratiometric Fura-2 based monitoring of [Ca^2+^]_i_ in freshly isolated split-opened ASDN with targeted deletion of P2Y2 and TRPV4 in mice to probe a role for purinergic signaling in mediating mechano-sensitive responses in ASDN cells. ATP application causes a reproducible transient Ca^2+^ peak followed by a sustained plateau. Individual cells of the cortical collecting duct (CCD) and the connecting tubule (CNT) respond to purinergic stimulation with comparative elevations of [Ca^2+^]_i_. Furthermore, ATP-induced Ca^2+^-responses are nearly identical in both principal (AQP2-positive) and intercalated (AQP2-negative) cells as was confirmed using immunohistochemistry in split-opened ASDN. UTP application produces elevations of [Ca^2+^]_i_ similar to that observed with ATP suggesting a dominant role of P2Y2-like receptors in generation of [Ca^2+^]_i_ response. Indeed, genetic deletion of P2Y2 receptors decreases the magnitude of ATP-induced and UTP-induced Ca^2+^ responses by more than 70% and 90%, respectively. Both intracellular and extracellular sources of Ca^2+^ appeared to contribute to the generation of ATP-induced Ca^2+^ response in ASDN cells. Importantly, flow- and hypotonic-induced Ca^2+^ elevations are markedly blunted in P2Y2 −/− mice. We further demonstrated that activation of mechano-sensitive TRPV4 channel plays a major role in the sustained [Ca^2+^]_i_ elevation during purinergic stimulation. Consistent with this, ATP-induced Ca^2+^ plateau are dramatically attenuated in TRV4 −/− mice. Inhibition of TRPC channels with 10 µM BTP2 also decreased ATP-induced Ca^2+^ plateau whilst to a lower degree than that observed with TRPV4 inhibition/genetic deletion. We conclude that stimulation of purinergic signaling by mechanical stimuli leads to activation of TRPV4 and, to a lesser extent, TRPCs channels, and this is an important component of mechano-sensitive response of the ASDN.

## Introduction

It is recognized that the distal part of renal nephron (also termed the aldosterone-sensitive distal nephron, or ASDN), which includes the connecting tubule (CNT) and the cortical collecting duct (CCD), is responsible for the final regulation of electrolyte (Na^+^, K^+^, Ca^2+^), water and acid-base balance. Abnormal regulation and/or dysfunction of the transporting systems in the ASDN are linked to a number of pathological states associated with changes in the circulating plasma volume and electrolyte imbalance [1]–[3].

Dynamic changes in renal tubular flow and fluid composition can be sensed by the cells of the ASDN [4], [5]. Indeed, substantial experimental evidence suggests that increases in tubular flow regulate Na^+^-reabsorption and K^+^-secretion [5]–[7]. It is also proposed that the ASDN cells respond to these environmental changes by increasing [Ca^2+^]_i_ [5]. Less is known about the molecular mechanisms and sources responsible for these Ca^2+^ elevations. An important physiological role of the primary cilium in flow-mediated cellular responses has been recently proposed [4]. Mutations in both PKD1 and PKD2 genes result in functional defects of the primary cilium accounting for all cases of autosomal dominant polycystic kidney disease [4]. However, intercalated cells (IC), which are devoid of primary cilium, respond to flow changes with comparable increases in [Ca^2+^]_i_ as principal cells (PC) [8].

Transient receptor potential (TRP) channels are known to participate in cellular adaptations to a variety of environmental stimuli, including thermo-sensation, chemo-sensation, mechanical forces etc (reviewed in [9]). Several TRP channels, including TRPC3, TRPC6, and TRPV4 can be detected with immunohistochemistry in the native ASDN cells and ASDN-derived cultured lines [4], [10]–[12]. Activation of these channels mediates Ca^2+^ entry from the extracellular medium, thus, possibly contributing to the elevation of [Ca^2+^]_i_ in response to mechanical stimuli [9]. Consistent with this, a role for TRPV4 in flow-mediating K^+^-secretion in the CCD has been recently proposed [13].

ATP is constitutively released from the ASDN cells [14], [15]. The mechanism is not fully elucidated but a critical role of the Connexin 30 (Cx30) hemi-channel as a conduit of ATP secretion has been recently suggested [16]. The physiological importance of the local purinergic signaling in controlling water-electrolyte transport in the ASDN has been unequivocally demonstrated using genetically modified mice. Mice lacking the P2Y2 receptor have salt-resistant hypertension and facilitated renal Na^+^ reabsorption [17], [18]. Mice lacking Cx30 hemi-channel have impaired ATP release in the ASDN and, as a consequence, develop salt-sensitive hypertension and impaired Na^+^ pressure-natriuresis [16]. Moreover, P2Y2 −/− mice have increased urine concentrating ability and enhanced responses to exogenous vasopressin [19].

ATP release in the ASDN is markedly augmented by changes in tubular flow rate as well as cell volume [14]. Locally released ATP can act in an autocrine/paracrine manner by targeting both ligand-operated P2X ion channels and G_q_-coupled P2Y receptors to regulate electrolyte and water transport in the ASDN (reviewed in [14], [15]). Stimulation of purinergic signaling in renal nephron, in turn, can increase [Ca^2+^]_i_ via PLC-IP_3_-mediated Ca^2+^ release from the endoplasmic reticulum (ER) [20], [21]. Moreover, apical ATP activates Ca^2+^-permeable TRPC3 channel to drive Ca^2+^ flux from tubular lumen in IMCD-3 cell line [22]. However, it is unclear whether ATP can also stimulate other TRP channels, such as mechano-sensitive TRPV4 in the ASDN.

In this study, we used freshly-isolated split-opened ASDNs to define the molecular mechanism of ATP-induced elevation of [Ca^2+^]_i_ and to test if purinergic signaling is involved in mechano-sensitivity of mammalian distal nephron. We found that ATP uniformly increases [Ca^2+^]_i_ in a PLC-sensitive manner in ASDN cells. Both extracellular and intracellular Ca^2+^ sources contribute to these Ca^2+^ elevations. Disruption of purinergic signaling in P2Y2 −/− mice markedly reduces cellular responses to mechanical stimulations. Importantly, ATP activates Ca^2+^-permeable TRPV4 and TRPC channels to elicit a sustained raise of [Ca^2+^]_i_ with TRPV4 having a major role. We conclude that activation of purinergic signaling by mechanical stimuli reciprocally contributes to the mechano-sensitivity by activating Ca^2+^-permeable TRPV4 channel.

## Materials and Methods

### Materials and animals

All chemicals and materials were from Sigma (St. Louis, MO), VWR (Radnor, PA), and Tocris (Ellisville, MO) unless noted otherwise and were of reagent grade. Animal use and welfare adhered to the NIH Guide for the Care and Use of Laboratory Animals following a protocol (#HSC-AWC-10-069) reviewed and approved by the Institutional Laboratory Animal Care and Use Committee of the University of Texas Health Science Center at Houston. For experiments, male C57BL/6 mice (purchased from Charles River Laboratories, Wilmington, MA), P2Y2 −/− (backcrossed and inbred into the C57BL/6 background; described in detail earlier [18], [23]), and TRPV4 −/− in a C57BL/6 background [24] 6–8 weeks old, were used. Animals were maintained on standard rodent regimen (PURINA, #5001) and had free access to tap water.

### Tissue isolation

**Figure 1:**
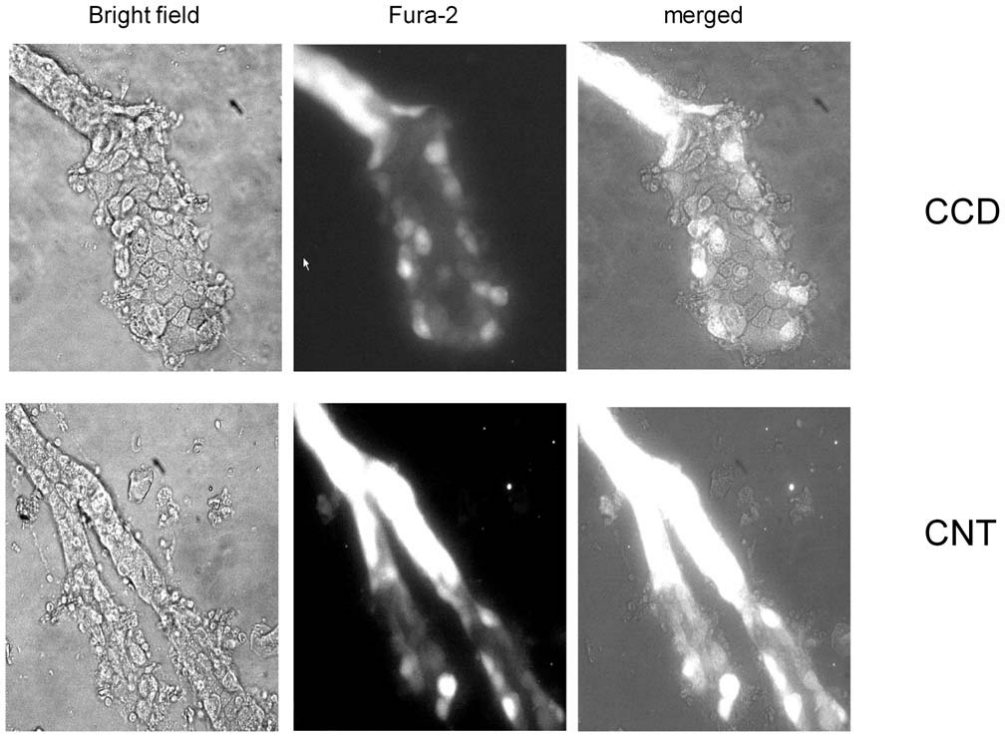
Ca^2+^-imaging in aldosterone-sensitive distal nephron. Representative micrographs of split-opened cortical collecting duct (top raw) and connecting tubules (bottom raw) after loading with Fura-2 taken with bright-field illumination (left column), 380 nm excitation (middle column), and the merged image (right column).

The procedure for isolation of the aldosterone-sensitive distal nephrons (ASDNs) containing the connecting tubule and the cortical collecting duct suitable for electrophysiology and Ca^2+^-imaging has been described previously [17], [25], [26]. Briefly, mice were sacrificed by CO_2_ administration followed by cervical dislocation and the kidneys were immediately removed. Kidneys were cut into thin slices (<1 mm) with slices placed into ice-cold physiologic saline solution buffered with HEPES (pH 7.4). The ASDN was identified as merging of CNT into CCD (see also Figure 1) and was mechanically isolated from cortical sections of kidney slices by micro-dissection using watchmaker forceps under a stereomicroscope. Isolated ASDN was attached to 5×5 mm cover glass coated with poly-L-lysine. A cover-glass containing ASDN was placed in a perfusion chamber mounted on an inverted Nikon Eclipse Ti microscope and perfused with room temperature HEPES buffered (pH 7.4) saline solution. ASDNs were split-opened with two sharpened micropipettes, controlled with different micromanipulators, to gain access to the apical membrane. The tubules were used within 1–2 hr of isolation.

### [Ca^2+^]_i_ measurements

Intracellular calcium levels were measured in cells of the split-opened ASDNs using Fura-2 fluorescence ratiometric imaging as described previously [12], [27]. Split-opened ASDNs were loaded with Fura-2 by incubation with 2 µM Fura-2/AM in a bath solution for 40 min at RT. Subsequently, the ASDNs were washed and incubated for an additional 10–15 minutes prior to experimentation. The ASDNs were then placed in an open-top imaging study chamber (Warner RC-10) with a bottom coverslip viewing window and the chamber attached to the microscope stage of an InCa Imaging Workstation (Intracellular Imaging, Inc.). Cells were imaged with a 20× Nikon Super Fluor objective and regions of interest (ROIs) drawn for individual cells. The Fura-2 fluorescence intensity ratio was determined by excitation (an average for ∼300 msec) at 340 nm and 380 nm and calculating the ratio of the emission intensities at 511 nm in the usual manner every 5 seconds. We observed no significant Fura-2 bleaching and minimal Fura-2 leakage at both wavelengths during experiments. The changes in the ratio are reported either as an index of changes in intracellular calcium [28] or converted to intracellular Ca^2+^ concentrations using the calibration methods as we have done before [29].

Experimental traces from individual cells were inspected visually prior to acceptance. For analysis, traces with initial F_340_/F_380_ ratio from 0.18 to 0.20 (which corresponds to ∼100–140 nM of [Ca^2+^]_i_) were selected and the baseline values were subtracted for generation of average time course for multiple cells. Typically, 3–5 individual ASDNs from an average of 3 mice were used for each experimental set.

### Immunohistochemistry

Split-opened tubules were fixed with 4% paraformaldehyde in PBS (pH = 7.4) for 20 min at RT. After fixation the samples were permeabilized by addition of 0.1% Triton in PBS for 5 min and washed in PBS 3 times for 5 min. Nonspecific staining was blocked with 10% normal goat serum (Jackson Immunoresearch, USA) in PBS for 30 min at RT. After washing with PBS (3 times for 5 min) the samples were incubated for 1.5 hr at RT in dark with anti-aquaporin 2 labeled with ATTO-550 (1∶100 dilution; Alomone labs) in 1% serum+0.1% Triton in PBS. After washing with PBS (3 times for 5 min) the samples were stained with 4′,6-diamidino-2-phenylindole (DAPI) (300 nM concentration, Calbiochem, San Diego, CA, USA) to visualize nuclei. Subsequently the samples were dehydrated, and mounted with permanent mounting media (Thermo Scientific, Pittsburg, PA, USA). Labeled tubules were examined with an inverted Nikon Eclipse Ti fluorescent microscope using a 40× Plan-Fluor oil-immersion (1.3 NA) objective. Samples were excited with 405 and 561 nm laser diodes and emission captured with a 16-bit Cool SNAP HQ^2^ camera (Photometrics) interfaced to a PC running NIS elements software.

### Solutions

Typical bath solution was (in mM): 150 NaCl, 5 mM KCl, 1 CaCl_2_, 2 MgCl_2_, 5 glucose and 10 HEPES (pH 7.4). All reagents were applied by perfusing the experimental chamber at 1.5 ml/min. To test the effect of elevated flow on [Ca^2+^]_i_, the rate of perfusion was instantly increased from 1.5 ml/min (∼15 mm H_2_O) to 15 ml/min (∼80 mmH_2_O). The linear velocity of flow increased from ∼0.13 cm/sec to 1.3 cm/sec under these conditions. For experiments testing the effect of hypotonic cell swelling, the isotonic solution contained (in mM) 100 NaCl, 5.4 KCl, 0.5 MgCl_2_, 0.4 MgSO_4_, 3.3 NaHCO_3_, 1.0 CaCl_2_, 10 HEPES, 5.5 glucose, 90 mannitol, pH 7.4, with osmolarity of 305 mOsm/L. The hypotonic solution contained (in mM): 100 NaCl, 5.4 KCl, 0.5 MgCl_2_, 0.4 MgSO_4_, 3.3 NaHCO_3_, 1.0 CaCl_2_, 10 HEPES, 5.5 glucose, pH 7.4, with osmolarity of 220 mOsm/L.

### Data analysis

All summarized data are reported as mean ± SEM. Data from before and after treatment within the same experiment were compared using the paired *t*-test. Data from different experiments were compared with a Student's (two-tailed) *t*-test or an One-Way ANOVA as appropriate. P≤0.05 was considered significant.

## Results

### Activation of purinergic signaling uniformly increases [Ca^2+^]_i_ in ASDN cells

Previous studies suggested that luminal application of ATP caused a prominent elevation of [Ca^2+^]_i_ in perfused CCD of rabbits and mice [14], [30], [31]. However, this approach has technical limitations in terms of precise separation of a fluorescent signal from a single cell. To circumvent this, we employed Fura-2 based Ca^2+^-imaging in freshly isolated split-opened murine ASNDs with multiple regions of interest (ROI) representing fluorescent signals from individual cells within a split-opened area. Figure 1 shows micrographs of a split-opened murine CCD (top) and a CNT with both branches split-opened (bottom) with bright-field illumination (left), fluorescent emission of FURA-2 with 380 nm excitation (middle), and the combined image (right). As is clear, this technique enables unequivocal separation of fluorescent signals from individual cells within a monolayer; and it allows direct pharmacological and mechanical manipulations with the apical surface of the cells.

**Figure 2:**
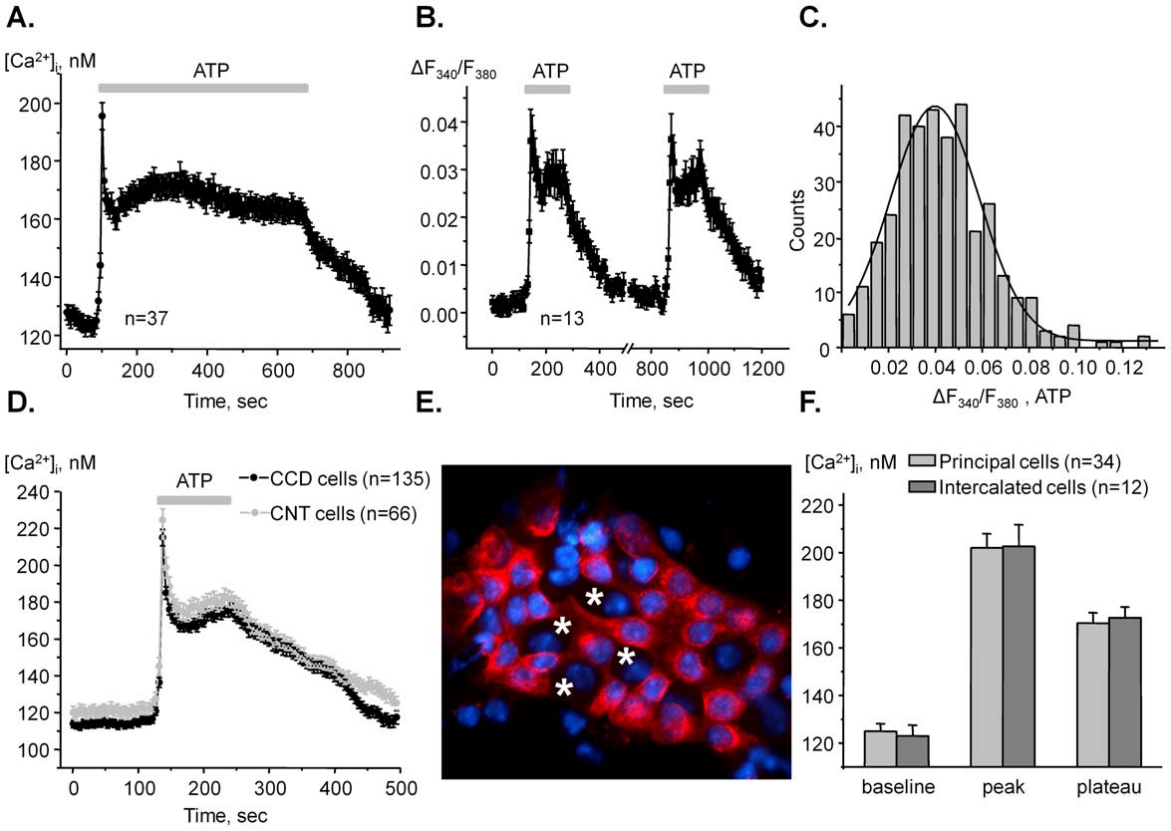
ATP application uniformly increases [Ca^2+^]_i_ in both PC and IC of ASDN. (**A**) The average time course of [Ca^2+^]_i_ changes in individual cells of ASDN in response to 10 min application of 10 µM ATP (shown with bar on the top). (**B**) The average time course of relative changes in ΔF_340_/F_380_ ratio in response to consecutive 2 min ATP applications (shown with bars). (**C**) Histogram of the magnitudes of ATP-induced ΔF_340_/F_380_ peak values of single cells in the ASDN. (**D**) The average time courses of elevations of [Ca^2+^]_i_ in response to ATP for individual cells of CCD and CNT (similar to that shown in Figure 1). (**E**) A representative fluorescent micrograph of AQP2 expression (pseudocolor red) within a split-opened area of ASDN. The examples of AQP2-negative (intercalated) cells are indicated with a white asterisk. Nuclear DAPI staining is also shown (pseudocolor blue). (**F**) Summary graph of [Ca^2+^]_i_ comparison for AQP2-positive (PC) and AQP2-negative (IC) cells similar to that shown in 2F in the resting condition (baseline) and during stimulation with 10 µM ATP (both peak and plateau values are reported).

We first aimed to quantify the ATP-induced Ca^2+^-responses in ASDN cells. Figure 2A documents the average time-course of changes in [Ca^2+^]_i_ in individual cells in response to 10 min application of 10 µM ATP. Purinergic stimulation caused a rapid (<20 sec rise time) Ca^2+^-spike followed by a sustained plateau with no obvious decline after 10 min of ATP application. [Ca^2+^]_i_ returned to basal levels upon ATP removal. Quantitatively, ATP increases [Ca^2+^]_i_ to 197±4 nM in the peak followed by 165±4 nM during the sustained plateau from the baseline of 123±3 nM (n = 37). We next tested if repetitive 2 min ATP stimulations elicit reproducible rises of [Ca^2+^]_i_ in ASDN cells. As is clear from the average time course of changes in F_340_/F_380_ ratio in Figure 2B the peak and the plateau of Ca^2+^ response were not attenuated during the second application.

It is well known that ASDN has two distinct cell types: principal cells (PC) and intercalated cells (IC). Several studies also point out that cells of CCD and CNT might posses different expression/activity of electrolyte transporting systems [32], [33]. Therefore, we next tested if this causes heterogeneity in ATP-induced Ca^2+^ responses. We first generated a histogram of the peak magnitude (over 350 responses analyzed) of ATP-induced [Ca^2+^]_i_ elevations in ASDN cells (Figure 2C). We expected to obtain a multimodal Gaussian distribution in the case if purinergic signaling is different between cell populations. However, as can be seen in Figure 2C, the distribution is clearly unimodal. This supports the idea that activation of purinergic signaling increases [Ca^2+^]_i_ to a similar extent in all ASDN cells.

We further address this by testing if ATP elicits similar Ca^2+^ responses in CNT and CCD cells. For analysis, we reasonably assumed that cells located upstream to the tubule bifurcation belong to the connecting tubule and cells located downstream to the merging point belong to the cortical collecting duct (see Figure 1). Figure 2D contains superposition of the average time courses of elevations of [Ca^2+^]_i_ in response to ATP for CCD (n = 135) and CNT (n = 66) cells. As can be seen, ATP-induced Ca^2+^-responses are not different between CNT and CCD cells.

**Figure 3:**
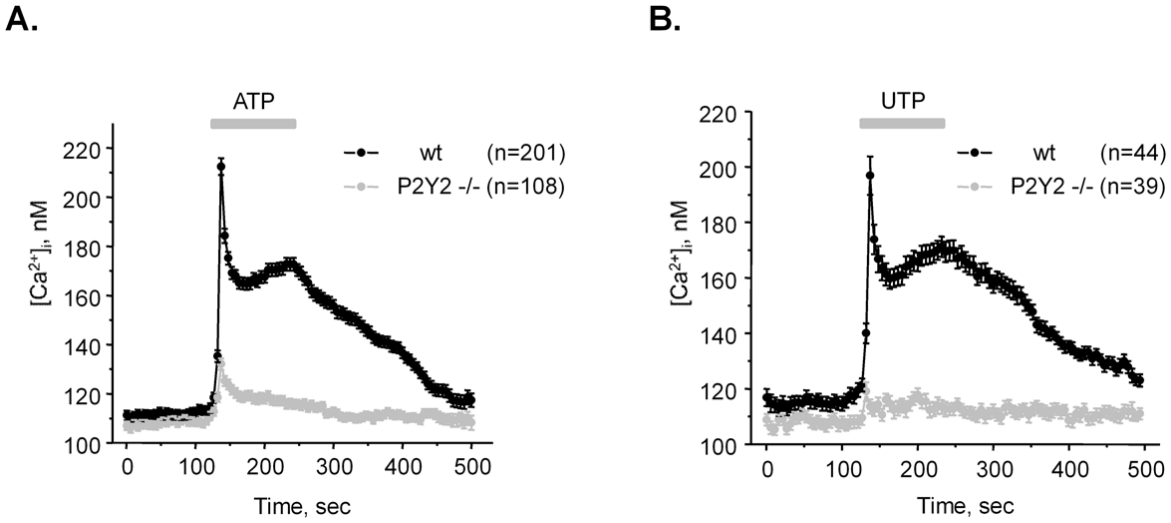
Genetic deletion of P2Y2 receptors abolishes Ca^2+^ transients in response to purinergic stimulation of ASDN cells. The average time course of changes in absolute [Ca^2+^]_i_ values in response to 2 min application of 10 µM ATP (**A**) and 10 µM UTP (**B**) for individual cells of ASDN from wild type (black) and P2Y2 −/− (light gray) mice, respectively. ATP and UTP application is shown as a bar on the top of each graph.

We next directly probed a role of purinergic signaling in principal and intercalated cells. For this, we quantified ATP-induced elevations of [Ca^2+^]_i_ from individual cells and then stained ASDNs with the specific marker of principal cells, AQP-2, for discrimination between cell types. As demonstrated by a representative micrograph (Figure 2E), PC (AQP2-positive) and IC (AQP2-negative) can be easily distinguished within a split-opened area. Figure 3F contains the summary graph of comparison of the baseline (125±3 nM and 123±4 nM) and the ATP-induced Ca^2+^ peak (202±6 nM and 203±9 nM) and the plateau (170±4 nM and 173±4 nM) for identified PC (n = 34) and IC (n = 12), respectively.

Overall, we concluded that activation of purinergic signaling with ATP induces reproducible uniform elevations of [Ca^2+^]_i_ in both principal and intercalated cells of ASDN.

### ATP stimulates P2Y2 receptors to increase [Ca^2+^]_i_ in ASDN cells

Previous studies by us and others proposed a dominant role for P2Y2 receptor as a sensor for ATP in the distal nephron [17], [23], [26], [31]. To test if stimulation of P2Y2 receptors accounts for elevations in [Ca^2+^]_i_ in ASDN cells we next monitored changes in [Ca^2+^]_i_ in response to 2 min applications of 10 µM ATP (Figure 3A) and 10 µM UTP (Figure 3B) for wild type and P2Y2 −/− mice. Both agonists induced a comparative rise of [Ca^2+^]_i_. Furthermore, genetic deletion of P2Y2 receptors decreased the amplitude of ATP-induced Ca^2+^ peak by more than 70% and UTP-induced Ca^2+^ peak by more than 90% (Figure 3). These results unequivocally suggest that P2Y2 receptors are central in mediating purinergic signal in the ASDN cells.

### Dysfunction of purinergic signaling impairs mechano-sensitive elevations of [Ca^2+^]_i_ in the ASDN

**Figure 4:**
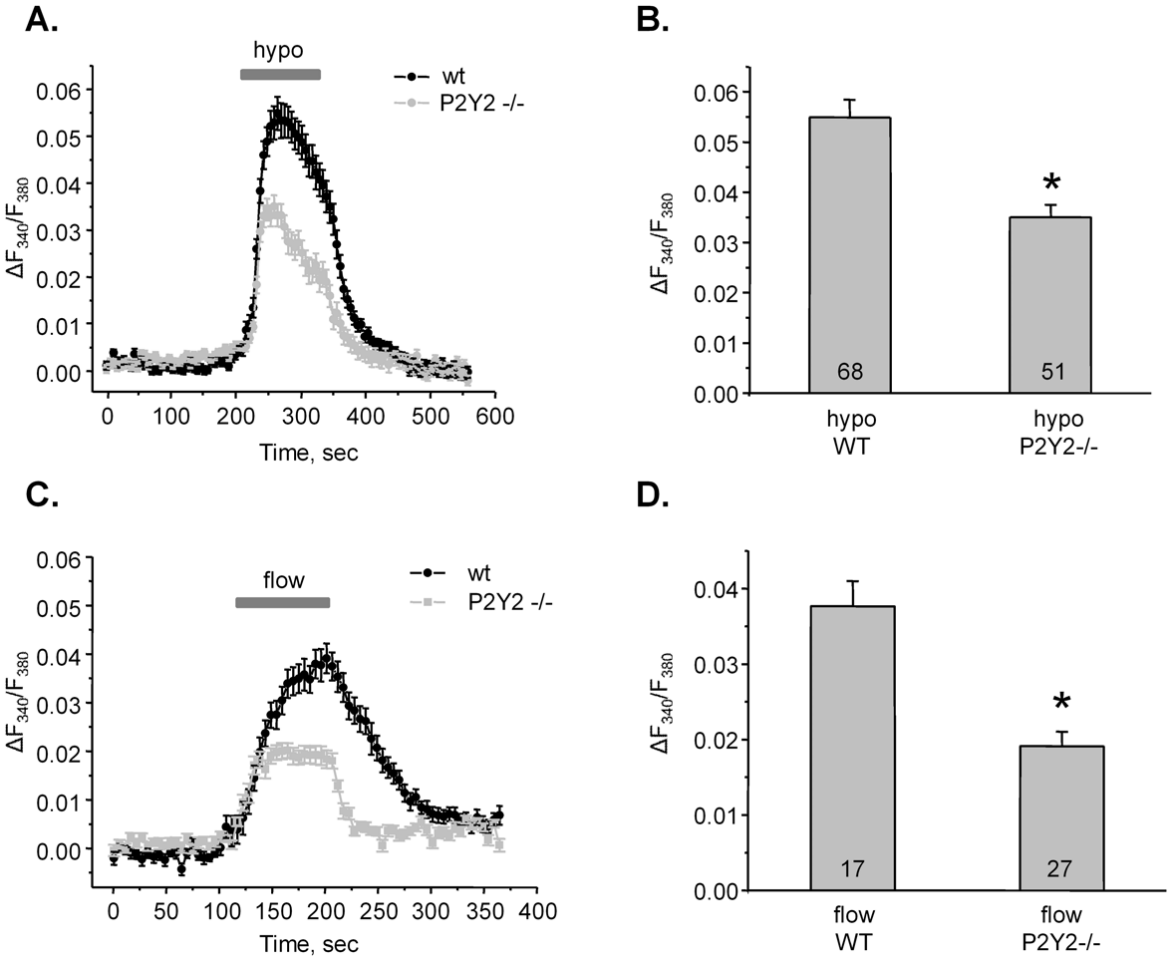
Disruption of purinergic signaling compromises mechano-sensitive responses in ASDN cells. (**A**) The average time course of relative changes in ΔF_340_/F_380_ in response to 2 min of hypotonic (hypo) media application (shown with bar on the top) for individual cells of ASDN isolated from wild type (wt, black) and P2Y2 −/− (light gray) mice. (**B**) Summary graph of the ΔF_340_/F_380_ peak changes in response to hypotonic media application for wild type and P2Y2 −/− mice. * - significant decrease versus hypo WT. (**C**) The average time course of relative changes in ΔF_340_/F_380_ in response to elevated flow (shown with bar on the top) for individual cells of ASDN from wild type (black) and P2Y2 −/− (light gray) mice. (**D**) Summary graph of the magnitudes of high flow-induced Ca^2+^ spikes for wild type and P2Y2 −/− mice. * - significant decrease versus flow WT.

Mechanical stimulation augments ATP release from ASDN cells [16]. Thus, we next probed whether purinergic signaling contributes to the elevations of [Ca^2+^]_i_ in response to changes in tubular flow and osmolarity. Figure 4A documents the average time course of changes in the fluorescent ratio in response to application of hypotonic (hypo) solution (220 mOsm) in individual cells of ASDN of wild type and P2Y2 −/− mice. Recall, genetic deletion of P2Y2 receptors disrupts purinergic Ca^2+^ signaling in the ASDN cells (see Figure 3). As is clear from the average time course in Figure 4A and the summary graph in Figure 4B, purinergic dysfunction results in decreased cellular [Ca^2+^]_i_ transient in response to hypotonic media. The mean ΔF_340_/F_380_ peak was 0.055±0.04 (n = 65) and 0.035±0.02 (n = 51) for wild type and P2Y2 −/− mice, respectively.

We next quantified the relative elevations in [Ca^2+^]_i_ in response to increases in the linear velocity of flow from 0.13 cm/sec to 1.3 cm/sec in wild type and P2Y2 −/− mice (Figure 4C, D). As shown for the average time course in Figure 4C and summarized in Figure 4D, flow induces marked sustained elevation of [Ca^2+^]_i_ and genetic deletion of P2Y2 receptors significantly blunts this response. The mean ΔF_340_/F_380_ was 0.038±0.003 (n = 17) and 0.019±0.002 (n = 27) for wild type and P2Y2 −/− animals, respectively.

Of note, flow-induced Ca^2+^ signals have identical initial rising phase but different steady state value in ASDN cells from wild type versus P2Y2 −/− animals. We can speculate that elevated flow stimulates initial Ca^2+^ entry from extracellular medium. Mechanical stimulation triggers ATP release which, in turn, contributes to the elevated [Ca^2+^]_i_. Therefore, we next aimed to determine the molecular determinants responsible for ATP-induced elevations of [Ca^2+^]_i_.

### ATP increases [Ca^2+^]_i_ in a PLC-dependent manner in murine ASDN cells

**Figure 5:**
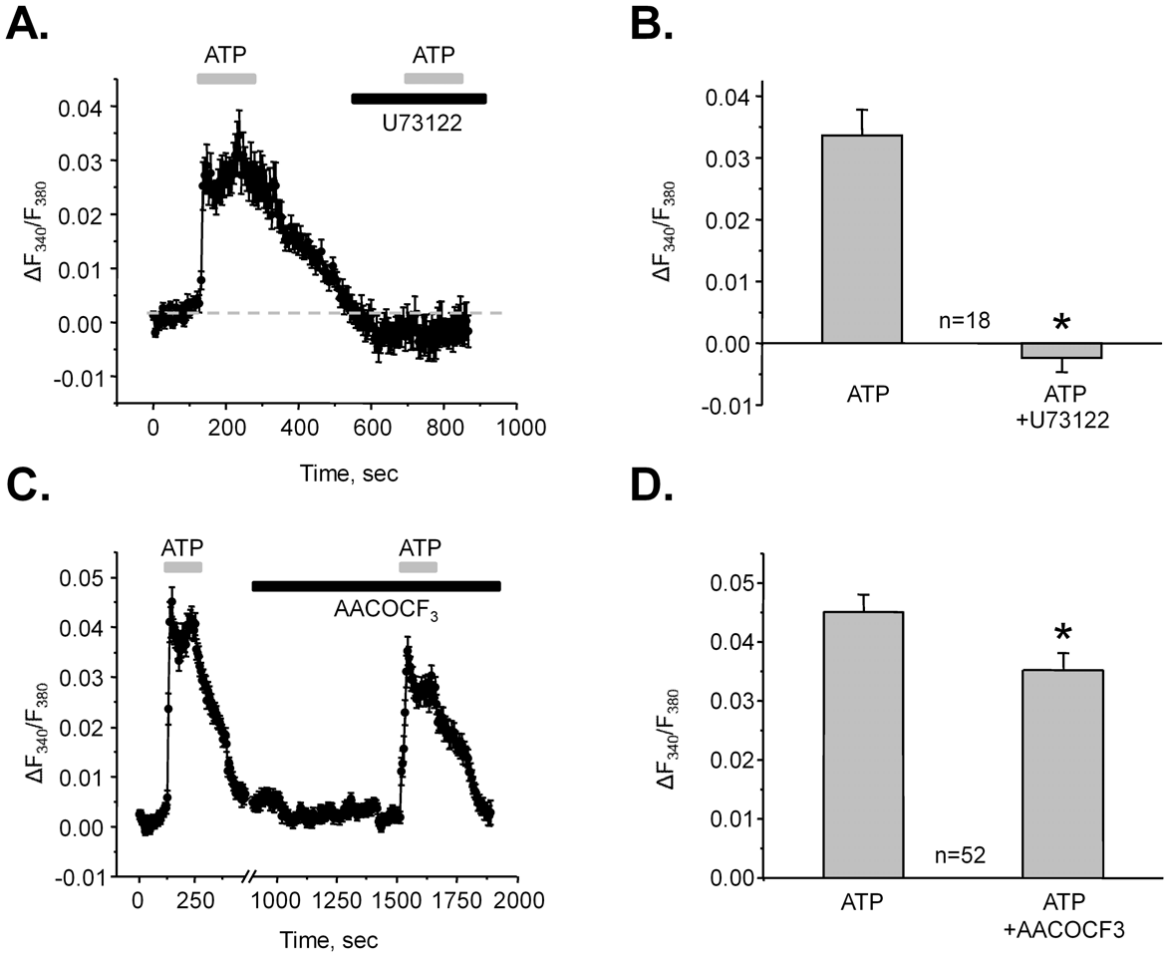
ATP increases [Ca^2+^]_i_ in a PLC-dependent manner. (**A**) The average time course of relative changes in ΔF_340_/F_380_ in response to 2 min ATP applications (shown with gray bar on the top) for individual cells of ASDN in the absence and presence of a PLC inhibitor, U73122 (black bar). (**B**) Summary graph of the ATP-induced changes in ΔF_340_/F_380_ in the control and after PLC inhibition. * - significant decrease versus ATP. (**C**) The average time course of relative changes in ΔF_340_/F_380_ in response to 2 min ATP applications (shown with gray bar on the top) for individual cells of ASDN in the absence and presence of a PLA inhibitor, AACOCF3 (black bar). (**D**) Summary graph of the ATP-induced changes in ΔF_340_/F_380_ in the control and after PLA inhibition. * - significant decrease versus ATP.

Our results in Figure 3 point to a dominant role of P2Y2 receptors in generation of ATP-induced Ca^2+^-response in ASDN cells. Stimulation of P2Y2 receptors typically leads to PLC activation and elevation of [Ca^2+^]_i_ via IP_3_-mediated mechanism. Thus, we next tested a role of PLC in generation of ATP-induced Ca^2+^-response. Figure 5A documents the average time course of the changes in [Ca^2+^]_i_ during ATP application alone and in the presence of a PLC inhibitor, U73122 (10 µM). As summarized in Figure 5B, PLC inhibition abolishes ATP-induced Ca^2+^ transients (ΔF_340_/F_380_ = 0.034±0.04 in the control and ΔF_340_/F_380_ = −0.002±0.002 in the presence of U73122, n = 18). Moreover, treatment with U73122 decreases basal Ca^2+^ levels (Figure 5A). This is consistent with our previous observations that paracrine ATP release from ASDN cells exerts inhibitory tone on ENaC-mediated Na^+^-reabsorption via PLC stimulation [17], [26], [34].

Elevations of [Ca^2+^]_i_ in response to ATP might also involve arachidonic acid (AA) signaling in innermedullary collecting duct cells [35]. Moreover, activation of purinergic signaling can lead to prostaglandin synthesis (mostly PGE2), thus, attenuating the vasopressin-mediated water transport in the distal nephron (reviewed in [15]). Therefore, we next assessed a role of PLA2 in generation of ATP-induced Ca^2+^-response in the ASDN (Figure 5C, D). Inhibition of PLA2 with AACOCF3 (30 µM for 10 min) modestly but significantly decreases ATP-induced Ca^2+^ responses: ΔF_340_/F_380_ = 0.045±0.002 in the control and ΔF_340_/F_380_ = 0.035±0.002 after AACOCF3 pre-treatment (n = 52). Therefore, the results in Figure 5 suggest that application of ATP increases [Ca^2+^]_i_ in a PLC-dependent manner and ATP-induced stimulation of PLA2 contributes to this response.

### ATP increases [Ca^2+^]_i_ using both intracellular and extracellular Ca^2+^ sources

**Figure 6:**
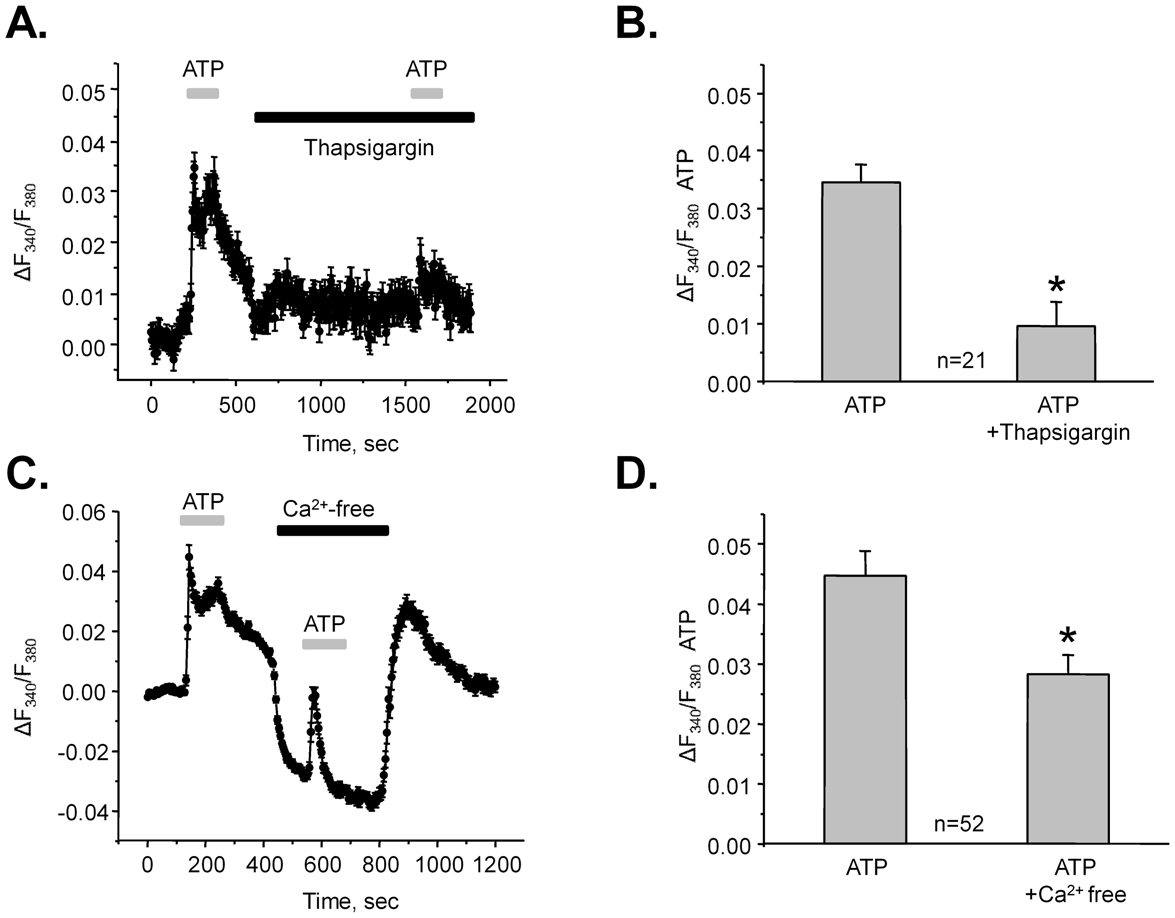
Extracellular and intracellular Ca^2+^ sources account for ATP-evoked [Ca^2+^]_i_ elevations. (**A**) The average time course of relative changes in ΔF_340_/F_380_ in response to 2 min ATP applications (shown with a gray bar on the top) for individual cells of ASDN in the absence and presence of a Ca^2+^-pump SERCA inhibitor, thapsigargin (black bar). (**B**) Summary graph of the ATP-induced changes in ΔF_340_/F_380_ in the control and after SERCA inhibition. * - significant decrease versus ATP. (**C**) The average time course of relative changes in ΔF_340_/F_380_ in response to 2 min ATP applications (shown with gray bar on the top) for individual cells of ASDN in the control and in Ca^2+^-free extracellular media (black bar). (**D**) Summary graph of the ATP-induced changes in ΔF_340_/F_380_ in the control and after extracellular Ca^2+^ removal. * - significant decrease versus ATP.

Stimulation of PLC in response to purinergic activation can lead to the Ca^2+^ release from the ER. Figure 6A documents the average time course of relative changes of [Ca^2+^]_i_ in response to ATP in the control and after 10 min pre-treatment with 2 µM Thapsigargin (TG) to inhibit the Ca^2+^-pump SERCA of the ER. As summarized in Figure 6B, ATP-induced Ca^2+^ responses were markedly attenuated after TG treatment. The amplitude of relative Ca^2+^ elevations was ΔF_340_/F_380_ = 0.035±0.002 in the control and ΔF_340_/F_380_ = 0.010±0.003 after TG (n = 21).

These results suggest that activation of purinergic signaling is capable of eliciting a Ca^2+^ response after depletion of intracellular Ca^2+^ stores. We hypothesized that activation of Ca^2+^-permeable ion channels on the plasma membrane during purinergic stimulus may contribute to the elevated [Ca^2+^]_i_. Therefore, we next quantified ATP-induced Ca^2+^-responses in Ca^2+^-free extracellular media (buffered with 5 mM EGTA). Ca^2+^ removal markedly decreased basal [Ca^2+^]_i_ which was returned to a control value after perfusing with standard solution (Figure 6C). Importantly, this maneuver had a small inhibitory effect on the ATP-evoked initial Ca^2+^ spike: ΔF_340_/F_380_ = 0.044±0.004 and ΔF_340_/F_380_ = 0.028±0.03 (n = 52) in the control and in Ca^2+^-free media, respectively (Figure 6C, D). However, it abolished the plateau component of the Ca^2+^-signal. These results support the idea that activation of Ca^2+^-permeable ion channels accounts for the sustained elevation of [Ca^2+^]_i_ in response to purinergic stimulation.

### Activation of TRP channels contributes to the purinergic signaling in the ASDN

**Figure 7:**
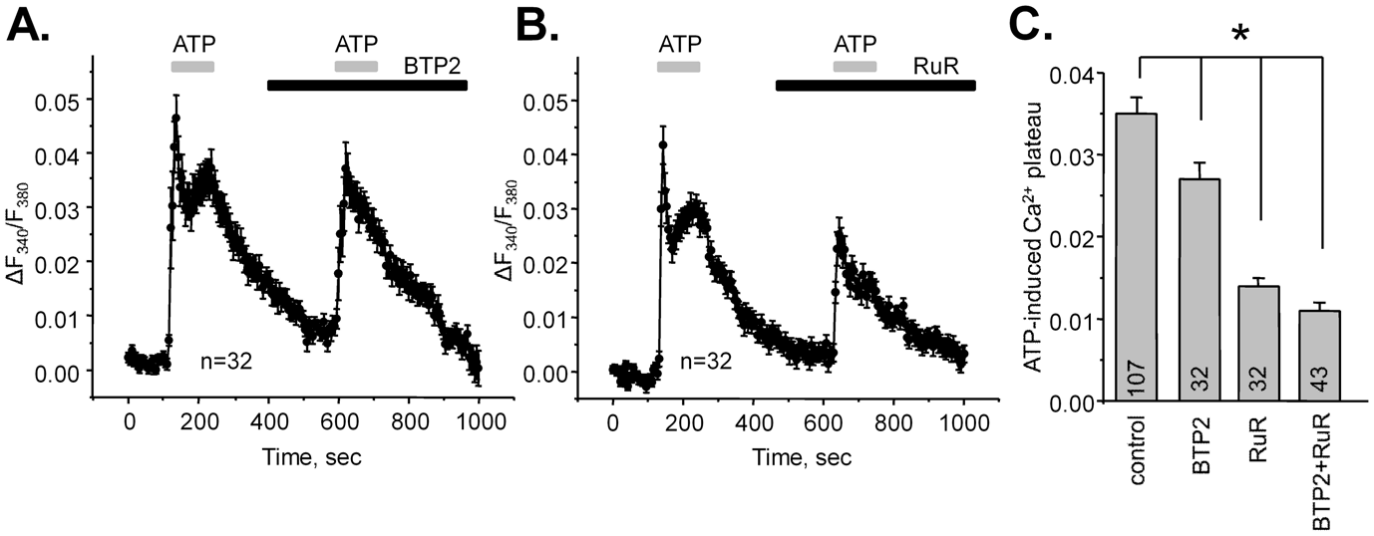
Ca^2+^-permeable TRPV and TRPC channels account for sustained elevation of [Ca^2+^]_i_ in response to ATP. The average time course of relative changes in ΔF_340_/F_380_ in response to 2 min ATP applications (shown with gray bar on the top) for individual cells of ASDN in the absence and presence (shown with black bar) of a TRPC channels inhibitor, BTP2 (**A**) and a TRPV channels inhibitor, RuR (**B**). (**C**) Summary graph of ATP-induced sustained elevation of [Ca^2+^]_i_ (plateau) in the control, after inhibition of TRPC with BTP2, after inhibition of TRPV with RuR, and after application of both inhibitors (BTP2+RuR). * - significant decrease versus control.

Several Ca^2+^-permeable channels can be detected with immunohistochemistry in the ASDN cells and ASDN-derived cultured lines [4], [11], [12]. These are TRPC3, TRPC6, and TRPV4. We next tested if activation of these channels contributes to the generation of [Ca^2+^]_i_ plateau in response to purinergic stimulation. Figure 7 contains the average time course of changes in fluorescence ratio after ATP application in the control and after inhibition of TRPC channels with 10 µM BTP2 (YM-58483) (Figure 7A), and after inhibition of TRPV channels with 1 µM Ruthenium Red (RuR) (Figure 7B). The summary graph on Figure 7C documents the steady-state elevation of [Ca^2+^]_i_ (plateau) in response to ATP in the control, after application of BTP2, RuR, and both inhibitors together (BTP2+RuR). As can be seen, inhibition of both TRPC and TRPV channels attenuated the sustained elevation of Ca^2+^ in response to ATP application with RuR having the greater effect.

**Figure 8:**
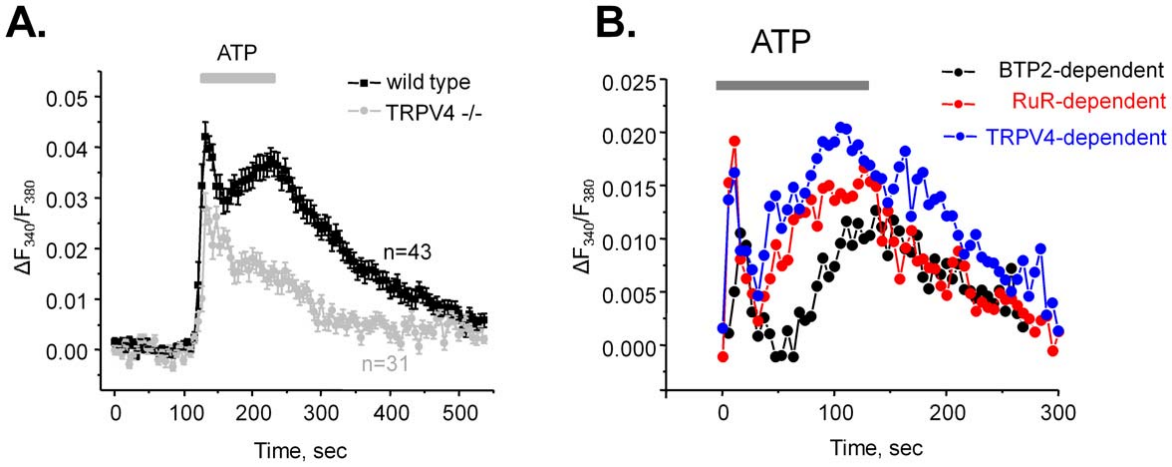
TRPV4 is critical for the ATP-induced Ca^2+^-plateau. (**A**) The average time course of relative changes in ΔF_340_/F_380_ in response to 2 min ATP application (shown with bar on the top) for individual cells of ASDN from wild type (black) and TRPV4 −/− (gray) mice. (**B**) Summary graph of relative contributions of TRPV4 and TRPCs in ATP-induced [Ca^2+^]_i_ response. For this, the values of ΔF_340_/F_380_ during second ATP application from Figures 7A, B, and 8A were subtracted from the corresponding values during the first ATP application.

The stronger RuR inhibition of ATP-induced Ca^2+^-elevations suggests a major role of TRPV4 channels in generation of the plateau of ATP-induced Ca^2+^ responses. To further test this, we next assessed the actions of ATP on [Ca^2+^]_i_ in mice lacking TRPV4 channels (TRPV4 −/− mice). Figure 8A contains the average time courses of ATP-induced Ca^2+^ responses for wild type and TRPV4 −/− mice. As is clear, genetic deletion of TRPV4 dramatically decreases the amplitude of the plateau. Importantly, the magnitude of this inhibition closely resembles the effect of RuR on ATP-induced [Ca^2+^]_i_ (Figure 7B). For better quantification of the relative contribution of TRPV4 and TRPC channels during activation of purinergic signaling we generated a graph of BTP2-, RuR-, and TRPV4-dependent component of Ca^2+^ response (Figure 8B). For this, we subtracted values of F_340_/F_380_ ratio during the second ATP response (after application of a respective inhibitor or during genetic deletion of TRPV4) in Figures 7A, B and 8A from those during the first ATP response (in the control).

Interestingly, we have also attempted to investigate a role for TRPP2 channel which is known to utilize TRPV4 to form a heteromeric mechano-sensitive molecular sensor in the cilium [36]. Unexpectedly, a TRPP2 blocker, amiloride, caused a reversible dose-dependent inhibition of both the initial transient ATP-induced Ca^2+^ spike and the sustained plateau (data not shown). This may suggest that amiloride, in addition to inhibiting TRPP2 channels, possesses non-specific inhibitory actions on P2Y receptors. This remains to be tested further in future studies.

**Figure 9:**
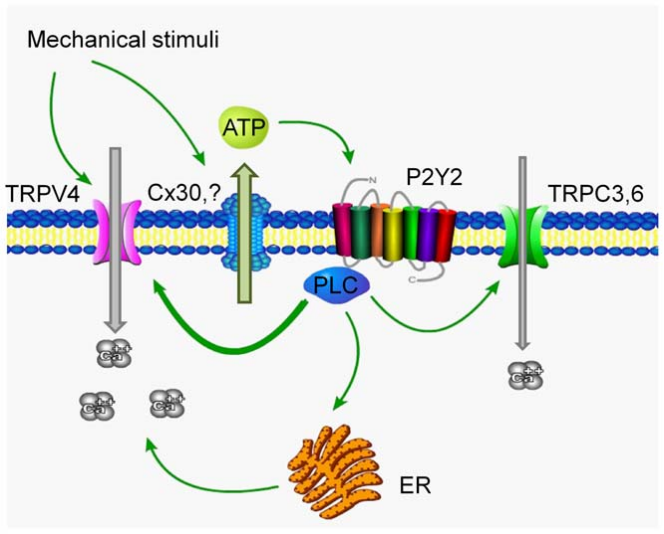
Principal scheme of the contribution of purinergic signaling to mechano-sensitivity in the aldosterone-sensitive distal nephron.

Overall, Figure 9 summarizes an involvement of purinergic signaling in [Ca^2+^]_i_ signalization in response to mechanical stress. Mechanical forces lead to activation of a mechano-sensitive TRPV4 channel. At the same time, ATP is released from the cells where Cx30 hemi-channels most likely mediate this process. ATP targets P2Y2 receptors and stimulates PLC in the same (autocrine action) or neighboring cells (paracrine action). Activation of PLC triggers Ca^2+^ release from the ER and also stimulates Ca^2+^-permeable mechano-sensitive TRPV4 and, to a lesser extent, TRPCs channels to further potentiate mechano-sensitive elevations in [Ca^2+^]_i_ in ASDN cells.

## Discussion

This study establishes a reciprocal link between mechano-sensitivity and paracrine purinergic signaling in native aldosterone-sensitive distal nephron. We argue here that locally released ATP during mechanical stimulation of ASDN is essential for the activation of mechano-sensitive Ca^2+^ permeable channels, such as TRPV4, which, in turn, augments cellular responses to mechanical stimuli. ATP failed to evoke a sustained raise of [Ca^2+^]_i_ in mice lacking the TRPV4 channel (Figure 8A). Recall, TRPV4 −/− mice have no flow dependence of Na^+^ and K^+^ transport in the CCD [13]. Moreover, we directly demonstrated that Ca^2+^ responses to elevated flow and hypotonicity are markedly diminished when purinergic signaling is compromised in P2Y2 −/mice (Figure 4).

In this study we performed careful characterization of a role for purinergic signaling in regulation of [Ca^2+^]_i_ in ASDN cells. We report that ATP evokes a rapid Ca^2+^-spike followed by a sustained plateau (Figure 2A) and that repetitive ATP applications elicit reproducible raises in [Ca^2+^]_i_ without evidence of desensitization (Figure 2B). In contrary, it was shown that luminal ATP fails to induce a raise of [Ca^2+^]_i_ in perfused CCD of rabbit when applied consequently [30]. We are not sure what may cause this discrepancy but the earlier study used 10 times higher ATP concentration (100 µM vs. 10 µM used here) which can potentially cause receptor desensitization. However, the reproducibility of Ca^2+^ transients in response to luminal ATP application (100 µM) from whole perfused murine CCDs was also demonstrated [37].

Interestingly, we found that ATP elicits identical Ca^2+^ responses in CNT and CCD cells (Figure 2D). Several groups provided experimental evidence that CNT might possess a higher rate of sodium and potassium transport than that of CCD [32], [33]. We and others previously demonstrated that luminal ATP inhibits ENaC-mediated sodium reabsorption in the murine distal nephrons [17], [26], [31]. It remains unclear if ATP plays a similar role in CCD and CNT assuming possible differences in electrolyte transport rates of sodium transport in these tubular segments.

We have also found that purinergic signaling is similar in both principal and intercalated cells. A critical role of intercalated cells in local ATP release in ASDN via Cx30-dependent mechanism was recently proposed [16]. Moreover, there is a functional coupling between BK-dependent potassium secretion and ATP release from intercalated cells [38]. Therefore, paracrine purinergic signaling might play an important role by orchestrating Na^+^ reabsorption and K^+^ secretion in electrically uncoupled principal and intercalated cells.

We found that ATP and UTP increases [Ca^2+^]_i_ to a similar extent in ASDN cells suggesting that that activation of P2Y2 receptors plays a major role in mediating [Ca^2+^]_i_ elevations in response to purinergic stimulation (Figure 3). Indeed, genetic deletion of P2Y2 receptors nearly abolished both ATP-mediated and UTP-mediated Ca^2+^ responses. This is consistent with a critical role of P2Y2 receptors in controlling ENaC-mediated Na^+^-reabsorption in the ASDN as we us [23], [26] and others [31] reported previously. The residual small elevations of [Ca^2+^]_i_ in response to ATP in P2Y2 −/− mice are most likely mediated by other P2Y receptors, such as P2Y1 and/or P2Y6, which are also expressed in the connecting tubule and collecting duct of mammals [14], [39]–[42]. However, it is unlikely that P2X receptors contribute to the response since inhibition of PLC abolishes ATP-induced elevations in [Ca^2+^]_i_ (Figure 5).

P2Y2 receptors can be expressed at both apical and basolateral sides of cells in ASDN (reviewed in [14]). Despite the fact that we applied ATP from the luminal side of the split-opened ASDN, the “back-leak” of the nucleotide to the basolateral side can occur. Indeed, we have been successful to stimulate basolateral V2 receptors with AVP to modulate ENaC activity in split-opened ASDNs [25]. However, basolateral ATP elicited much smaller elevations of [Ca^2+^]_i_ compared to the apical ATP application in perfused murine CCDs [37]. In our case this response might be even smaller considering lower concentration of ATP on the basolateral side due to the “back-leak” effect. Therefore, while we recognize a possible role of basolateral side, the luminal P2Y2 receptors most likely play a major role in generation of the ATP-induced Ca^2+^ response in our experiments.

We documented that both intracellular and extracellular Ca^2+^ sources contribute to the elevations of [Ca^2+^]_i_ in response to ATP (Figure 6). Specifically, initial transient rise in [Ca^2+^]_i_ was mediated by the Ca^2+^ release from the ER whereas activation of Ca^2+^-permeable membrane channels was responsible for the sustained [Ca^2+^]_i_ plateau. Importantly, inhibition of PLC abolished ATP-mediated changes in [Ca^2+^]_i_ suggesting that the activation of Ca^2+^-permeable channels occurs in a PLC-dependent manner (Figure 5A). This is in agreement with previous observations that PLC inhibition disrupts regulation of electrolyte transport by ATP in the ASDN [26]. In addition, activation of purinergic signaling can counteract vasopressin mediated water transport in the collecting duct [15], [19], [35]. The possible mechanism involves COX-dependent prostaglandin release suggesting an involvement of AA metabolism. We confirmed a role of PLA2 in generation of the Ca^2+^-signal in response to ATP, however, this contribution was modest at best (Figure 5C, D).

It is known that mechanical stimulation triggers ATP release from many epithelia including that of ASDN [14], [16], [43]. There is a controversy of whether flow-induced ATP release contributes to the mechano-sensitive [Ca^2+^]_i_ signal. It was reported that inhibition of P2 receptors with suramin does not prevent flow-mediated Ca^2+^ response in perfused CCDs of rabbit [30] but does so in cultured MDCK cells [44]. In our study, we took advantage of genetically modified mice with disruption of P2Y2 receptors to probe if dysfunction of purinergic signaling impedes mechano-sensitive properties of the murine ASDN. We show here that elevations of [Ca^2+^]_i_ in response to elevated flow and hypotonic media are strongly attenuated in mice lacking P2Y2 receptors (Figure 4). We are not certain of why pharmacological inhibition of P2 receptors with suramin [30] does not reproduce the effect we observed in P2Y2 −/− mice (Figure 4C, D). However, a critical role of luminal ATP signaling in flow response in perfused thick ascending limb (TAL) was also demonstrated using P2Y2 −/− mice [21].

An important finding of this study is that activation of purinergic signaling stimulates Ca^2+^-permeable mechano-sensitive TRPV4 channels (Figures 7, 8) to further augment cellular responses to elevated flow. We have previously shown strong expression of TRPV4 at the luminal membrane of murine CCD [12]. Interestingly, basolateral expression of TRPV4 in CCD of mice and rats was also shown using immuhohistochemistry [45]. However, there is no experimental evidence as yet demonstrating functional activity of TRPV4 on the basolateral membrane. In contrast, luminal but not basolateral application of a TRPV4 agonist, 4αPDD, augmented flow-dependent K^+^ secretion and Na^+^ reabsorption in the CCD [13]. Here, we documented a reduce in flow-mediated elevations of [Ca^2+^]_i_ in mice with compromised purinergic signaling (P2Y2 −/− mice). A recent study suggested that luminal ATP can stimulate TRPC3 in cultured IMCD-3 cells and that this channel is critical for sustained elevation of [Ca^2+^]_i_ in response to ATP [22]. We also documented a contribution of TRPC channels to ATP-induced Ca^2+^ response in native ASDN (Figures 7, 8). However, the role of these channels was limited with TRPV4 being a major contributor to the sustained [Ca^2+^]_i_ plateau. We can speculate that IMCD-3 cells, which are typically not exposed to variations in flow, switch their phenotype from expression of a flow-sensor TRPV4 to canonical G-protein activated TRPCs. Interestingly, it is also proposed that activation of TRPC3 channels has a role in the apical-to-basolateral Ca^2+^ flux [27]. It raises an intriguing idea that Ca^2+^ permeable channels of ASDN, in addition to having a signaling role, might also account for Ca^2+^ reabsorption at this segment. Further studies are necessary to test this possibility.

An important aspect of mechano-sensitivity of the ASDN is how cells sense the mechanical forces. Strong experimental evidence argues for a critical role of central cilium as a reporter of velocity of tubular fluid flow. For instance, cellular responses to flow are blunted in *orpk* mice which have impaired structure/function of primary cilium [46]. Mutations in polycystins (PC1 and PC2), which are localized to the cilia, are associated with development of polycystic kidney disease [4]. Moreover, dysfunction of PC1 and PC2 results in inability of kidney cells to sense mechanical stresses [4]. However, intercalated cells which have no primary cilia respond to elevated flow by increasing [Ca^2+^]_i_ as it happens in principal cells [30]. We also did not observe heterogeneity in cellular responses to elevated flow and hypotonicity, though, we did not directly discriminate cell types (Figure 4). Furthermore, TRPP2 (PC2) by itself fails to respond to mechanical stimuli [47] but requires TRPV4 to gain mechano-sensitivity [36]. Consistent with this, a recent study suggested that Ca^2+^ permeable TRPV4 serves as a flow sensor for flow-dependent K^+^ secretion and Na^+^ reabsorption in the CCD [13]. Interestingly, TRPV4 −/− mice do not develop any symptoms of PKD despite the impaired flow sensing in the kidney [13], [24]. We can speculate that the major role of TRPV4 is to mediate cellular responses to mechanical stimuli by elevating [Ca^2+^]_i_, whereas TRPP2 (PC2), while participates in this process, is mainly involved in the development of PKD.

In summary, we defined a role of purinergic signaling in regulation of mechano-sensitivity in the distal nephron. In contrast to the TAL where purinergic signaling accounts for the mechano-sensitive response [21], paracrine ATP is an important modulator of the Ca^2+^-permeable TRP channels, such as TRPV4, which underlie the mechano-sensitive properties of the ASDN. Disruption of purinergic signaling in P2Y2 −/− mice does not lead to PKD development but does impair mechano-sensitivity in the distal nephron. Interestingly, growing experimental evidence suggests that cysts formation also leads to a dysfunction of purinergic signaling [48]. Thus, the exact role of paracrine ATP in PKD pathology is waiting to be determined.
